# Irinotecan plus raltitrexed as first-line treatment in advanced colorectal cancer: a phase II study

**DOI:** 10.1038/sj.bjc.6601713

**Published:** 2004-03-16

**Authors:** J Feliu, A Salud, P Escudero, L López-Gómez, C Pericay, C Castañón, M R López de Tejada, J M Rodríguez-García, M P Martínez, M Sanz Martín, J J Sánchez, M González Barón

**Affiliations:** 1 Medical Oncology Service, Hospital La Paz, P de la Castellana, 261-28046 Madrid, Spain; 2 Hospital Arnau Vilanova de Lérida, Spain; 3 Hospital Clínico Universitario de Zaragoza, Spain; 4 Hospital Virgen de la Salud de Toledo, Spain; 5 Hospital Santa Creu y San Pau de Barcelona, Spain; 6 Hospital General de Leon, Spain; 7 Hospital Punta de Europa de Algeciras, Spain; 8 Hospital Nuestra Señora de la Candelaria de Tenerife, Spain; 9 Hospital de Basurto, Spain; 10 Hospital Nuestra Señora de Alarcos de Ciudad Real; 11 Unidad de Estadística de la Universidad Autónoma de Madrid, Spain

**Keywords:** colorectal cancer, chemotherapy, raltitrexed, irinotecan, CPT-11

## Abstract

To evaluate the efficacy and toxicity of irinotecan (CPT-11) in combination with raltitrexed as first-line treatment of advanced colorectal cancer (CRC). A total of 91 previously untreated patients with advanced CRC and measurable disease were enrolled in this phase II study. The median age was 62 years (range 31–77); male/female 54/37; ECOG performance status was 0 in 50 patients (55%), one in 39 (43%) and two in two (2%). Treatment consisted of CPT-11 350 mg m^−2^ in a 30-min intravenous infusion on day 1, followed after 30 min by a 15-min infusion of raltitrexed 3 mg m^−2^. Measurements of efficacy included the following: response rate, time to disease progression and overall survival. Of the 83 evaluable patients valuable to objective response, there were five complete responses (6%) and 23 partial responses (28%), for an overall response rate of 34% (95% CI: 25.9–46.5%). In all, 36 patients (43%) had stable disease, whereas 19 (23%) had a progression. The median time to progression was 11.1 months and the median overall survival was 15.6 months. A total of 487 cycles of chemotherapy were delivered with a median of five per patient. Grade 3–4 WHO toxicities were as follows: diarrhoea in 13 patients (15%), nausea/vomiting in four (4%), transaminase increase in six (7%), stomatitis in two (2%), febrile neutropenia in three (3%), anaemia in five (6%) and asthenia in three (3%). The combination CPT-11–raltitrexed is an effective, well-tolerated and convenient regimen as front-line treatment of advanced CRC.

For more than 40 years, 5-fluorouracil (5FU) was the only cytotoxic agent with significant activity in advanced colorectal cancer (CRC). However, during the last decade, new cytotoxic agents, such as the topoisomerase I inhibitor irinotecan (CPT-11) or the platinum derivative oxaliplatin have demonstrated significant single-agent activity in the setting of first- and second-line chemotherapy. The value of the addition of CPT-11 to 5FU–LV, as first-line treatment for metastatic CRC, has been established in two large randomised trials (Douillard *et al*, 2000; Saltz *et al*, 2000). These trials demonstrated that combination chemotherapy significantly improved the response rate, median time to progression and overall survival in patients with advanced CRC (Douillard *et al*, 2000; Saltz *et al*, 2000). Alternatively, the addition of oxaliplatin to 5FU–LV significantly improved the overall response rate and median time to progression but not the median survival time (Giacchetti *et al*, 2000; de Gramont *et al*, 2000).

Although combined therapy regimens are an important development in the treatment of advanced CRC, they have a number of disadvantages. On the one hand, there is an increased toxicity, especially with the CPT-11–5FU–LV combination when 5FU is administered as a bolus injection (Ledermann *et al*, 2001; Rothenberg *et al*, 2001). On the other hand, these regimens require either repeated in-hospital administration or the use of infusion pumps, which may have a negative impact on the patient's quality of life. Accordingly, further investigation is needed into new regimens that are less toxic and more convenient for patients, while maintaining the same efficacy as the previous ones.

Raltitrexed is a specific inhibitor of thymidylate synthase. This enzyme has a fundamental role in the *de novo* synthesis of the nucleotide thymidine triphosphate, which is essential for DNA synthesis. At a dose of 3 mg m^−2^, it is active in a variety of tumours such as breast, pancreatic or refractory ovarian cancers (Cunningham *et al*, 1994), but it is in colorectal tumours where it shows the best activity (Van Custem *et al*, 2002). Several phase III studies performed in patients with advanced CRC demonstrated that response rates and survival were similar to that of the combination 5FU–LV (Cunningham *et al*, 1995; Cocconi *et al*, 1998; Maughan *et al*, 2002).

However, in another randomised trial, a survival advantage in favour of patients treated with conventional 5FU–LV (Pazdur and Vicent, 1997) was seen even when the response rate was similar. The administration as a short 15-min intravenous infusion every 3 weeks adds value to the efficacy and toxicity profile of raltitrexed. In fact, in a study which compared the patients' preferences between raltitrexed and other 5FU-based regimens with regard to side-effect attributes and administration attributes, 91% of the patients expressed a preference for the former treatment (Young *et al*, 1999).

CPT-11 and raltitrexed have different mechanisms of action and experimental studies have shown a sequence-specific synergistic cytotoxicity. Synergistic effects were demonstrated with a short-term exposure to SN-38 (the CPT-11 active metabolite), followed by raltitrexed. However, the reverse sequence, longer exposure or co-exposure had an antagonistic effect (Aschele *et al*, 1998).

Accordingly, the CPT-11–raltitrexed combination is expected to be at least as effective as the CPT-11–5FU–LV combination, but more convenient and less toxic for the patient. A phase I study with the combination CPT-11–raltitrexed demonstrated that this regimen is active and associated with an acceptable toxicity. The recommended dose for phase II studies was 350 mg m^−2^ CPT-11 and 3 mg m^−2^ raltitrexed (Ford *et al*, 2000).

The purpose of this multicentre phase II study is to assess the efficacy and safety of the CPT-11–raltitrexed combination as first-line treatment in patients with advanced CRC.

## PATIENTS AND METHODS

### Patient population

From September 1999 to January 2001, 91 patients with recurrent or metastatic CRC were included. They all had at least one lesion histologically confirmed as adenocarcinoma. Patients who had received prior adjuvant 5FU-based chemotherapy were eligible if they had remained free of disease for at least 6 months after the completion of adjuvant therapy. Patients with operable metastatic disease were excluded from the study. Other inclusion criteria were: (1) performance status ⩽2, according to the Eastern Cooperative Oncology Group (ECOG) scale; (2) life expectancy of at least 3 months; (3) adequate haematological parameters (that is a granulocyte count ⩾2 × 10^9^ l^−1^ and platelets >100 × 10^9^ l^−1^); (4) adequate hepatic function, that is serum bilirubin <1.25 times the upper normal limit, glutamic oxaloacetic transaminase values (SGOT) and glutamic pyruvic transaminases (SGPT) <2.5 times the upper normal limit in the absence of hepatic metastases or <5 times the upper normal limit in the presence of metastasis; (5) adequate renal function, that is a serum creatinine value ⩽1.25 times the upper normal limit and creatinine clearance >65 ml min^−1^.

Patients with any prior chemotherapy for advanced disease, brain or meningeal metastases, or a history of any other malignancy, were excluded, except in cases of basal cell carcinoma or *in situ* cervical carcinoma adequately treated. Patients provided written informed consent according to directives of local ethical committees.

All patients had measurable disease, as defined by the presence of at least one bidimensionally measurable lesion by computed tomography scan. Pleural effusion, ascites, osteoblastic lesions or previously irradiated lesions were not accepted as measurable disease. Patients who had received radiotherapy were eligible if there was at least one measurable lesion outside the radiation field.

### Treatment plan

The study regimen consisted of CPT-11 350 mg m^−2^ in a 30-min intravenous infusion on day 1, followed after 30 min by a 15-min infusion of raltitrexed 3 mg m^−2^.

CPT-11 was administered according to the guidelines used for CPT-11 monotherapy, including recommendations for using atropine and loperamide. Routine antiemetic prophylaxis with a 5-hydroxytryptamine-3-receptor antagonist was used. Courses were repeated every 21 days for a minimum of three per patient, unless progressive disease was detected. Patients with partial response or stable disease remained on chemotherapy until progression or the appearance of unacceptable toxicity.

Patients were assessed for toxicity before each course and graded according to standard WHO criteria (WHO, 1979). Complete blood counts were obtained before the beginning of each course. Therapy was delayed for 1 week if the neutrophil count was <1.5 × 10^9^ l^−1^ or the platelet count <100 × 10^9^ l^−1^ or for significantly persisting non-haematological toxicity. Therapy was definitely discontinued if toxicity persisted after a 2-week delay. In case of grade 3 or 4 haematological toxicity, the dose of all drugs was decreased by 25 or 50%, respectively. If grade 2 or 3 diarrhoea or stomatitis occurred, the dose was reduced by 25 or 50%, respectively; grade 4 diarrhoea or stomatitis led to treatment withdrawal.

The Cockcroft–Gault formula (Cockcroft and Gault, 1976) was used to calculate creatinine clearance before each cycle. If creatinine clearance was between 55 and 65 ml min^−1^, the next cycle was given 4 weeks later, and if it was between 25 and 54 ml min^−1^, the dose of raltitrexed was reduced by 50% and the next cycle given 4 weeks later. If it was <25 ml min^−1^, the treatment was interrupted.

### Pretreatment and follow-up studies

Patients underwent the following baseline evaluations: full clinical history, physical examination, performance status assessment, haematological and biochemical profiles (including CEA level), chest X-ray and computed tomography scan of the chest and abdomen at baseline. Additional imaging investigations were performed if clinically indicated. Computed tomography scan was repeated every three courses to assess the objective response. At the end of chemotherapy, all clinical, laboratory and imaging studies were repeated and patients underwent follow-up examination every 2 or 3 months until death.

### Toxicity and response criteria

Toxicity for each course was recorded and graded according to WHO scales (WHO, 1979). For toxicity analysis, the worst data for each patient across all courses were used. All patients who received at least three cycles of therapy were considered for objective tumour response. Response was evaluated by using the WHO guidelines (WHO, 1979). All patients who receive at least one cycle were evaluable to toxicity.

A complete response required the total disappearance of all initially detected tumours in two observations not less than 4 weeks apart, with any evidence of new areas of malignant disease. A partial response was defined as a reduction of at least 50% in the sum of the products of the longest perpendicular diameter of all clearly measurable tumour masses, in two observations not less than 4 weeks apart, with no increase in the size of any lesion and no evidence of new lesions. Stable disease was defined as a decrease in total tumour size of less than 50% or a less than 25% increase in any measurable lesion. Progression was defined as a 25% increase in the size of any lesion, the appearance of new areas of malignant disease or performance status deterioration by more than one level.

Time to tumour progression was estimated by the product limit estimation from the date of the first course to the first evidence of disease progression. Global survival was calculated by the same method from the date of the first course until the date of death or last known follow-up.

### Statistical analysis

The primary end point was the response rate and the secondary objectives were the clinical benefit, survival and time to progression. Dose intensity was calculated by dividing the total mg m^−2^ of drug given by the number of weeks elapsed from the beginning of therapy to the end of the last cycle.

The sample size was designed to reject a response rate of less than 20%. According to the Fleming (1982) method, 19 patients were first included.

Alternatively, a planned sample size of 90 evaluable patients was chosen to better estimate the efficacy; 20% maximum width of the 95% confidence interval (CI) for an expected 35% overall response rate.

Univariable analysis was used to compare the response rate, survival and incidence of grade 3–4 toxicity between the different groups formed according to age, gender, ECOG performance status, adjuvant chemotherapy *vs* no adjuvant chemotherapy, liver metastasis *vs* no liver metastasis and number of metastatic sites. The Wilcoxon rank-sum test to compare the quantitative variables and the exact Fisher test for the percentages were used. Survival time and time to progression were calculated using the Kaplan–Meier's method. The Cox proportional hazard model was applied to survival dates.

## RESULTS

### Patient characteristics

A total of 91 patients with recurrent or metastatic CRC were entered into the study. The characteristics of these patients are shown in Table 1

**Table 1:** Patients characteristics

**Characteristic**	**No. (%)**
Age (mean and range)	62 (31–77)
	
*Sex*	
Male	54 (59%)
Female	37 (41%)
	
*ECOG performance status*	
0	50 (55%)
1	39 (43%)
2	2 (2%)
	
*Primary site*	
Colon	54 (59%)
Rectum	37 (41)
	
*Metastasis sites*	
Liver	65 (71%)
Lung	29 (32%)
Local abdominal mass	23 (25%)
Others	22 (24%)
	
*No. of metastatic sites*	
1	34 (37%)
2	25 (28%)
⩾3	32 (35%)
	
*Adjuvant chemotherapy*	
Yes	44 (48%)
No	47 (52%)

. The median age of the series was 62 years (range 31–77). In all, 54 patients were males (59%) and 37 were females (41%). There were 50 patients (55%) with ECOG-0 performance status, 39 (43%) with ECOG-1 and two (2%) with ECOG-2. In 54 patients (59%), the primary tumour was located in the colon and in 37 (41%) in the rectum. Synchronous metastatic disease was observed in 47 patients (52%), in 22 of which (24%) the primary tumour was not resected. Of the remaining patients, 44 (48%) presented metastases secondary to a tumour previously removed. Of these patients, 30 (33%) had previously received chemotherapy as adjuvant and 14 (15%) had received chemotherapy and radiotherapy. The liver was the predominant metastatic site (71%), and the median number of involved sites was two per patient.

A total of 487 cycles were given with a median of five cycles per patient (range 1–14). In all, 16 patients (18%) received less than three cycles of chemotherapy: eight (9%) due to a progression, four (4%) due to the patient's refusal, two (2%) moved to a different city and two (2%) due to death apparently not related to the neoplasia (one due to acute stroke and another due to acute myocardial infarction). The last eight patients (9%) were not evaluable for objective tumour response. In 12 patients (13%) treatment had to be delayed on some occasions due to the following reasons: an increase of transaminases in six, diarrhoea in three, neutropenia in one and at the patient's request in another two. The median dose intensity of CPT-11 and raltitrexed was 115 and 0.98 mg m^−2^ week^−1^, respectively. A total of 85 patients (85%) received 90% or more of the planned dose.

Second-line chemotherapy with oxaliplatin was given to 54% of those patients who progressed or relapsed.

### Tumour response and survival

Documented complete response was observed in five patients (6%) and partial response in 23 (28%), for an overall response rate of 34% (95% confidence interval (CI) 25.9–46.5%). In addition, 36 patients (43%) remained with a stable disease and 19 (23%) showed a progression (Table 2

**Table 2:** Therapeutic results in 83 patients

**Results**	**No. of patients (%)**
Complete response	5 (6%)
Partial response	23 (28%)
Stable disease	36 (43%)
Progressive disease	19 (23%)

Overall response 34% (95% CI: 25.9–46.5%).

). The progression free survival was 11.1 months. The median survival was 15.6 months. The actuarial 1-year survival was 58%. No relationship between response rate and site of metastases, number of metastatic sites, previous adjuvant chemotherapy, ECOG performance status, age or sex was observed. After analysing the relationship between these potential prognostic factors and the progression-free time or survival, the ECOG 0 patients were found to have a longer progression free time than those with an ECOG 1–2 status (13.3 *vs* 7.9 months; *P*<0.002). Similarly, a relationship between the ECOG performance status and the overall survival was found (21.4 months for ECOG 0 *vs* 8.3 months for ECOG 1–2; *P*<0.0001). The ECOG performance status remained as the only survival-related variable in the Cox multivariable regression model.

### Toxicity

Treatment was well tolerated generally. In total, 64 patients (70%) suffered some toxicity, usually grade 1–2. The main toxicities were gastrointestinal and haematological. In all, 16 patients (18%) developed grade 3–4 side effects (Table 3

**Table 3:** Toxicity per patient (WHO grades) during the whole trial

**Toxicity**	**Per patient (89)**			**Per cycle (487)**		
	**Grade 1–2**	**Grade 3**	**Grade 4**	**Grade 1–2**	**Grade 3**	**Grade 4**
Anaemia	24 (27%)	5 (6%)		60 (12%)	7 (1%)	
Thrombocytopenia	3 (3%)			3 (1%)		
Nausea/vomiting	59 (66%)	3 (3%)	1 (1%)	184 (38%)	10 (2%)	3 (1%)
Diarrhoea	32 (36%)	10 (11%)	3 (3%)	94 (19%)	7 (1%)	6 (1%)
Stomatitis	6 (7%)	2 (2%)		8 (2%)	2 (1%)	
Transaminases	26 (29%)	4 (4%)	2 (2%)	73 (15%)	7 (1%)	2 (1%)
Asthenia	27 (30%)	3 (3%)		109 (22%)	3 (1%)	
Alopecia	31 (35%)	6 (7%)	3 (3%)	99 (20%)	14 (3%)	16 (3%)
Cholinergic syndrome	20 (22%)			44 (9%)		

): 13 patients developed diarrhoea (15%), four had nausea/vomiting (4%), six a transaminase increase (7%), two stomatitis (2%), three febrile neutropenia (3%), five anaemia (6%) and three asthenia (3%). One patient suffered an episode of atrial fibrillation during the fourth cycle, which was terminated by using medical treatment. Four treatment-related hospital admissions were reported and no toxic deaths occurred. No relationships between the occurrence of grade 3–4 toxicity and the patient's ECOG performance status, age or sex were found.

## DISCUSSION

The recent introduction of new drugs against advanced CRC with distinct mechanisms of action has made it possible to achieve better results than those obtained so far with the administration of 5FU–LV. The combination of CPT-11 with 5FU–LV has not only led to a 15% increase in the response rate and a 3-month prolongation in the median progression-free time with respect to traditional 5FU–LV regimens, but has also resulted in a 2–3-month increase of median survival (Douillard *et al*, 2000; Saltz *et al*, 2000). However, this modest although significant improvement has been attained at the expense of increased toxicity. In fact, approximately one fourth of the patients receiving these regimens have grade 3–4 diarrhoea or grade 4 neutropenia (Douillard *et al*, 2000; Saltz *et al*, 2000). In addition, when administered as a bolus injection together with CPT-11, 5FU–LV has been found to increase the risk of toxic death associated with gastrointestinal toxicity or thromboembolic disorders (Rothenberg *et al*, 2001). It is thus necessary to explore other alternative regimens that are less toxic and more convenient for the patient, while maintaining or improving their efficacy. Our study should be considered from this perspective.

The results obtained from our series (with an overall response rate of 37%, a median progression-free time of 11.1 months and a median survival of 15.6 months) suggest that the studied regimen is effective for advanced CRC. Besides, its moderate toxicity, with only 15% of patients having grade 3–4 diarrhoea and 3% of patients with febril neutropenia, should be highlighted. These results seem to be comparable to those found with regimens combining 5FU–LV and CPT-11. However, given the lack of phase III head-to-head comparative trials with these regimens, caution is advised when making such comparisons, since differences may exist between our patients' characteristics and those of patients included in the above series (Douillard *et al*, 2000; Saltz *et al*, 2000). So, while they are similar in age, ECOG performance status and proportion of patients with liver metastases, some differences may exist in the percentage of patients receiving adjuvant chemotherapy (48% in our series *vs* 11% in the series of Saltz *et al*). The proportion of patients with three or more metastatic sites is also different (35% in our series *vs* 10% in the series of Saltz *et al vs* 15% in that of Douillard *et al* (2000). The prognostic relevance of this finding in patients with advanced CRC has recently been underlined (Douillard *et al*, 2000; Köhne *et al*, 2002).

In spite of the good results achieved in our series, it should be emphasised that the sequence that we used to administer the study drugs might not have been the most appropriate. The findings from *in vitro* studies suggest that the maximum synergism of action is obtained when there is a 24-h interval between administration of CPT-11 and raltitrexed (Aschele *et al*, 1998; Jackman *et al*, 1999). However, we have followed the schedule designed by Ford *et al* (2000) in which both drugs were given on the same day. In agreement with these authors, we consider that this administration approach could be more convenient for patients. This same regimen has been used as second-line therapy in a series of 52 patients with 5FU-resistant CRC, where a partial response rate of 13.5% was obtained with moderate toxicity (grade 3–4 diarrhoea, 23%; grade 3–4 neutropenia, 17%) (Aparicio *et al*, 2003). Other authors, however, have investigated the activity and tolerance of CPT-11 administration followed by raltitrexed 24 h later (Stevenson *et al*, 2001). In a phase II study including 46 patients, an overall response rate of 46% was achieved, with a median overall survival of 57 weeks. These results were obtained at the expense of a significant toxicity; grade 3–4 diarrhoea and grade 3–4 neutropenia were present in 26 and 20% of patients, respectively (Carnaghi *et al*, 2002). On the other hand, a phase I study addressed a CPT-11 schedule (days 1 and 8) in combination with raltitrexed (days 1 or 2) every 21 days. The CPT-11 recommended dose was 100 and 3 mg m^−2^ for raltitrexed on day 1. However, when raltitrexed was administered on day 2, these doses were not tolerated and a CPT-11 dose reduction to 75 mg m^−2^ was necessary. Overall responses that reached 23% in the 26 patients included were reported (Lewis *et al*, 2002). Alternatively, the bi-weekly administration of CPT-11 and raltitrexed has been examined in an attempt to increase the dose intensity. In a phase I/II trial including 20 patients, a partial response rate of 35% and a median survival of 13 months were achieved (Colucci *et al*, 2001). Results obtained from these studies appear similar to those found in our series; however, these schedules may be more inconvenient for patients, since they need to increase the number of hospital visits for treatment.

Other important issues to consider when assessing a chemotherapy regimen are the patients' preferences, the economic cost and its potential impact on the quality of life. In this regard, a study that analysed the patients' preferences with respect to the toxicity profile and the ease of administration showed that 91% of patients preferred raltitrexed to other 5FU-based regimens, such as those advocated by The Mayo Clinic, Gramont or Lokich (Young *et al*, 1999). Furthermore, a study analysing the treatment-related costs concluded that, although raltitrexed treatment-related costs were high, they were compensated for when savings attributable to its ease of administration were considered (Groener *et al*, 1999). Of greater concern, however, are the results of a study that compared the Lokich and Gramont regimens with raltitrexed.

Raltitrexed treatment was found to achieve comparable results to those from the Lokich and Gramont regimens as for response and survival rates, with costs that were similar to those from the Lokich regimen and almost half those derived from the Gramont regimen. Nevertheless, a greater toxicity, a lower quality of life in comparison with other regimens and an increase of treatment-related deaths were observed in raltitrexed-treated patients (Hale *et al*, 2002; Maughan *et al*, 2002). In this regard, a special attention is to be given to the patient's renal function before each raltitrexed cycle in order to prevent the occurrence of unexpected toxicities (Jansman *et al*, 2000). In fact, raltitrexed AUC may even double when creatinine clearance is lower than 65 ml min^−1^ (Judson *et al*, 1998). In the study by Hale *et al*, creatinine levels were not regularly documented, although routine assessment of creatinine clearance prior to each raltitrexed administration was specified in the protocol (Cunningham *et al*, 2002). In addition to these measures, it should be kept in mind that the prophylactic use of 5-HT3 antagonists and dexamethasone reduces the incidence and severity of nausea, diarrhoea, fever and asthenia associated with the administration of raltitrexed (Thomas, 2000).

Thus, although all these data suggest that raltitrexed-based combinations can improve the treatment acceptance by the patients over other schedules requiring more frequent hospital visits or the carrying of an infusion pump; further investigation is needed to evaluate its impact on the patient's quality of life.

In conclusion, our study results suggest that the combination of CPT-11 with raltitrexed is an active and moderately toxic regimen for first-line treatment of the advanced CRC. Accordingly, we reckon that it represents an attractive and convenient alternative for the treatment of these patients. A large randomised trial is needed to compare the efficacy of this regimen with the standard combination of CPT-11/5FU–LV in patients with advanced CRC.
